# Cost‐Effectiveness of Group‐Based Outpatient Physical Therapy After Total Knee Replacement: Results From the Economic Evaluation Alongside the ARENA Multicenter Randomized Controlled Trial

**DOI:** 10.1002/acr.24903

**Published:** 2022-09-12

**Authors:** Estela C. Barbosa, Vikki Wylde, Joanna Thorn, Emily Sanderson, Erik Lenguerrand, Neil Artz, Ashley W. Blom, Elsa M. R. Marques

**Affiliations:** ^1^ University of Bristol, Bristol Medical School Bristol UK; ^2^ University of Bristol, Bristol Medical School and NIHR Bristol Medical Research Centre Bristol UK; ^3^ University of Gloucestershire Gloucester UK

## Abstract

**Objective:**

To assess the cost‐utility and cost‐effectiveness of a group‐based outpatient physical therapy intervention delivered 6 weeks after primary total knee replacement (TKR) compared with usual care, alongside the Activity‐Orientated Rehabilitation Following Knee Arthroplasty (ARENA) multicenter, randomized, controlled trial.

**Methods:**

The economic analyses were performed from the perspective of the health and social care payer. We collected resource use for health and social care and productivity losses and patient outcomes for 12 months after surgery to derive costs and quality‐adjusted life years (QALYs). Results were expressed in incremental cost‐effectiveness ratios (ICERs), and incremental net monetary benefit statistics (INMBs) for a society willingness‐to‐pay (WTP) threshold of £20,000 per QALY gained, with sensitivity analyses to model specification and perspective.

**Results:**

The cost of the ARENA physical therapy classes was mean ± SD £179 ± 39 per patient. Treatment in the year following surgery cost was, on average, £1,739 (95% confidence interval [95% CI] –£742, £4,221) per patient in the intervention group (n = 89), which was an additional £346 (95% CI £38, £653) per patient compared with usual care (n = 91) (£1,393 [95% CI –£780, £3,568]). QALY benefits were 0.0506 higher (95% CI 0.009, 0.09) in the intervention group, corresponding to an additional 19 days in “perfect health.” The ICER for the intervention group was £6,842 per QALY gained, and the INMB was £665 (95% CI £139, £1,191), with a 92% probability of being cost‐effective, and no less than 73% in all sensitivity analysis scenarios.

**Conclusion:**

The addition of group‐based outpatient physical therapy classes to usual care improves quality of life and is a cost‐effective treatment option following TKR for a society WTP threshold of £20,000 per QALY gained.

## INTRODUCTION

Each year, around 110,000 people receive knee replacements in the UK (1, 2) as do an additional 10,000 in Sweden (3), 11,000 in Norway (4), and 790,000 in the US (5). Primary total knee replacements (TKRs) account for around 87% of all knee replacement surgeries in the UK. On average, patients are 70 years of age (interquartile range 64–77 years) (1). A small proportion of patients experience reduced mobility and persistent pain after knee replacement (6), which is associated with worse health‐related quality of life (QoL) (7). Physical therapy can improve short‐term outcomes after knee replacement (8), but provision is variable across the UK and often only when needed (9).

**SIGNIFICANCE & INNOVATIONS**

- We found that supplementing usual care with a novel 6‐week group‐based outpatient physical therapy intervention is a cost‐effective treatment option for the health and social care payer to offer all patients after primary total knee replacement for a society willingness‐to‐pay threshold of £20,000 per quality‐adjusted life year gained.
- Patients in the intervention arm had better quality of life in the year following surgery and spent, on average, an additional 19 days in “perfect health” compared with patients in the usual care group.
- Delivering the physical therapy intervention was relatively cheap (£179 per patient, on average). A year of care in the intervention group cost, on average, £346 more per patient compared with usual care.

The Activity‐Orientated Rehabilitation Following Knee Arthroplasty (ARENA) randomized controlled trial aimed to investigate whether providing a novel group‐based outpatient physical therapy intervention to all patients would be an effective and cost‐effective treatment option to optimize function in the longer term (10). The ARENA intervention led to a small improvement in function at 3 and 12 months after surgery, albeit below the minimum clinically important difference (10). The aim of this paper is to report the results of the cost–utility and cost‐effectiveness analyses performed alongside the ARENA trial.

## PATIENTS AND METHODS

### Overview of economic evaluation

We have conducted a 12‐month cost–utility analysis and a cost‐effectiveness analysis alongside the ARENA trial. The trial compared a group‐based outpatient physical activity intervention, offered to all patients after primary TKR, with usual care, which may or may not offer physical therapy to some (10). The primary analysis took a National Health System (NHS) plus Personal Social Services (PSS) (NHS + PSS) perspective, in accordance with National Institute for Health and Care Excellence (NICE) guidelines (11). The secondary analysis took a societal perspective on costs, which included private expenses and productivity losses. All analyses followed the prespecified health economics analysis plan (12) and study protocol (13).

### Trial design

The ARENA study was a multicenter, pragmatic, unblinded, superiority randomized controlled trial aiming to investigate whether providing a novel group‐based outpatient physical therapy intervention to all patients would be effective and cost‐effective. The trial design was informed by a systematic review (8), a survey of current practice (14), and a feasibility study (15). Clinical results of the ARENA trial are reported elsewhere (10).

### Patients

The ARENA trial recruited 180 patients waiting for TKR for osteoarthritis from 2 large orthopedic centers in Bristol, UK including Southmead Hospital, an NHS‐funded hospital at North Bristol NHS trust, and Emersons Green, a private independent treatment center. To relieve pressure in NHS hospitals, the NHS often contracts elective TKR surgeries to the private sector. All patients in the ARENA trial were NHS patients treated in these 2 different hospitals. All physical therapy received in the intervention and control arms was also funded by the NHS, regardless of hospital providing the surgery. The patients were randomized (n = 89 in the intervention group and n = 91 in the usual care group) and followed up at 3, 6, and 12 months by postal questionnaires. Patients completed a baseline questionnaire prior to surgery.

### Intervention and usual care

The ARENA intervention is an outpatient physical therapy intervention, consisting of 6 weekly 1‐hour group‐based physical therapy classes, starting 6 weeks after surgery. Classes were delivered in an NHS outpatient gymnasium by 2 physical therapists, or 1 physical therapist and a technician, on a weekly rolling basis. Classes could accommodate a maximum of 12 patients per class. Patients could join and leave the group freely so that at any one time the group would include patients who had undertaken differing numbers of sessions than each other. Patients completed an exercise circuit, consisting of 10 task‐related exercise stations and 2 individualized exercise stations. Physical therapists individualized exercises for each patient within a task‐oriented exercise circuit.

Usual care consisted of knee‐specific and functional advice and referral to outpatient physical therapy on a need‐specific basis, depending on the range of motion postsurgery or muscle weakness. Further details of the intervention and usual care are described in the protocol and clinical effectiveness papers (10, 13).

### Resource use for the economic evaluation

NHS costs included: 1) additional physical therapy received in hospital or in the community; 2) other therapies (such as hydrotherapy, instrument chiropraxis, or acupuncture); 3) hospital readmissions; 4) additional outpatient appointments or Accident and Emergency department attendances; and 5) medications. PSS costs included food‐at‐home services, home care help services, and special orthopedic equipment or house adaptations. In our societal perspective, we further included: 1) patient out‐of‐pocket health care and therapy expenses (if any); 2) lost income; and 3) productivity losses in terms of time off paid and unpaid work, time off usual activities, and time spent on informal care by a friend or relative.

### Outcome measures for the economic evaluation

We used the EuroQol 5‐domain, 5‐level (EQ‐5D‐5L) (16) questionnaire, a standardized and validated patient‐reported outcome instrument, for collecting health‐related QoL data and deriving quality‐adjusted life years (QALYs). The EQ‐5D‐5L consists of 1 question for each of the 5 dimensions, including mobility, self‐care, usual activities, pain/discomfort, and anxiety/depression. It allows outcomes for different clinical interventions to be directly comparable.

The Lower Extremity Functional Scale (LEFS) was the primary clinical outcome for the ARENA trial. The LEFS is a 20‐item patient‐reported outcome measure that produces a total score including questions on 4 different groups of activities, including hardest activities, moderately difficult activities, moderately easy activities, and easy activities. Each item ranges from 0 (extremely difficult) to 4 (no difficulty), and the total score ranges from 0 (high disability) to 80 (no disability). A minimum clinically important difference in LEFS is defined as a 9‐point difference or more (17).

### Data collection

Resources used in relation to the delivery of the intervention were recorded on study report forms. Physical therapists and physical therapy technicians also recorded time to prepare class, set up the gym, and clear up after class, as well as writing patient notes. These data enabled the estimation of the cost of the intervention.

Data on resource use were collected from patients at 3, 6, and 12 months, using postal resource‐use questionnaires (RUQs). Bespoke RUQs were designed by the research team (including orthopedic surgeons and physical therapists) in collaboration with a musculoskeletal patient and public involvement and engagement (PPIE) group. We further designed resource use logs for patients to track the use of resources prospectively and advised patients to refer to their logs when completing the RUQs. Data on outcomes (EQ‐5D‐5L scores [18] and LEFS) were collected prior to surgery (baseline) and at 3, 6, and 12 months postoperative. Resources used within 2 weeks of surgery were not collected, as randomization took place 2 weeks after surgery and no difference in resource use between groups within 2 weeks was expected.

### Patient and public involvement

The trial design and management was informed by a group of 9 patients in a PPIE group (19). Patients in the PPIE group informed the design of the RUQs.

### Dealing with missing data

We explored the patterns of missingness in the data and assumed data were not missing completely at random. We used multiple imputation methods (20), using chained equations, with 60 sets and predictive mean matching. Missing cost variables, utility scores, and LEFS scores were imputed at each time point (3, 6, and 12 months) and later aggregated. It was computationally not feasible to impute EQ‐5D scores by domain. Our imputation model included age, sex, hospital site, baseline utility, and LEFS scores.

### Valuing resource use to derive costs

In a micro‐costing approach, we used the Unit Costs of Health and Social Care to value staffing costs, using staff grades and time spent delivering the intervention (21). Other cost components were valued in a macro‐costing approach based on UK NHS reference costs (22) for community care and secondary care and the British National Formulary for medications (23). The costs associated with productivity losses and informal care were valued using a human capital approach and the Office for National Statistics–averaged gross weekly wages per age group. The cost associated with each resource‐use item was calculated by multiplying the units of the resource used in the 12‐month period by its unit cost, creating a measure of cost per year. All resources were valued in 2017–2018 Great British pounds (£).

### Valuing health states in the EQ‐5D‐5L to derive QALYs

We attached published UK societal utility tariffs for the EQ‐5D‐3L to the EQ‐5D‐5L response profiles using van Hout's crosswalk (18), as per the NICE position statement on valuation of the EQ‐5D‐5L (24). This produced a composite health‐related QoL score at each time point (2 weeks, and 3, 6, and 12 months after surgery) for each patient. These QoL or utility scores were treated as continuous variables, bound at a maximum of 1 (corresponding to “perfect health”), where 0 corresponds to death, and negative values were permitted for health states worse than death. We calculated accumulated QALYs gained per patient using the area‐under‐the‐curve approach, assuming a linear change between utility scores at each time point.

### Cost–utility and cost‐effectiveness analysis

We adjusted costs and QALYs for hospital site (trial stratification variable) and prespecified need‐predicting variables (age, sex, and comorbidities) as controls. The index for comorbidities was based on the count of simultaneous comorbidities per patient, in line with the literature (25). QALYs were further adjusted for utility at baseline and LEFS score at baseline (26). Costs and QALYs were not discounted due to the 12‐month time frame of the analysis.

We used seemingly unrelated regressions (SUR) to jointly estimate the differences in costs and outcomes between arms from baseline. The SUR methodology has the advantage of also calculating the correlation of residuals between costs and QALYs and testing if the 2 are independent or related. We then calculated the incremental cost‐effectiveness ratio (ICER) and the incremental net monetary benefit statistic (INMB), using a society willingness‐to‐pay (WTP) threshold of £20,000 per QALY, in accordance with NICE guidelines (27, 28). We plotted cost‐effectiveness acceptability curves for primary and secondary analyses, to illustrate the uncertainty surrounding the decision to adopt the intervention by indicating the probability that our group physical therapy intervention is cost‐effective over usual care for a range of societal WTP values.

All analyses were based on intention‐to‐treat; randomized participants were included in the economic analysis based on the group to which they were originally assigned, regardless of whether they received the allocated treatment or not.

### Uncertainty and sensitivity analyses

We addressed the uncertainties around our analysis methods and findings by conducting 1‐way deterministic sensitivity analyses. We recognize the cost of the intervention may vary in different settings and patient groups within the NHS. We created an optimistic scenario (where all physical therapy classes were attended by 12 patients) and a pessimistic scenario (where only 2 patients attended each physical therapy class), to understand the impact of class size on costs and cost‐effectiveness. We conducted a complete case analysis to handle the uncertainty arising from imputing missing data and examined patterns of missingness in the data. To address model uncertainty in the estimation of our costs and QALYs, we made adjustments using a second set of models. Models of type 2 included all variables in the first model plus ethnicity, employment status, alone living status, marital status, education, and Index of Multiple Deprivation deciles, as per the statistical analysis of clinical results (published elsewhere [29]). The trial received ethics approval from the National Research Ethics Committee Southwest‐Central Bristol (reference 14/SW/1144). All participants provided informed, written consent.

### Availability of data and material

The data sets generated during the current study will be available in the University of Bristol Research Data Repository (https://data.bris.ac.uk/data/). Access to the data will be restricted to ensure that data are only made available to bona fide researchers for ethically approved research projects, on the understanding that confidentiality will be maintained and after a Data Access Agreement has been signed by an institutional signatory.

## RESULTS

### Descriptive statistics

Table 1 shows patient demographic and socioeconomic characteristics by arm and overall. A total of 180 patients were randomized between March 2015 and March 2017. No major differences were observed between trial arms for all variables except marital status.

**Table 1:** Participants’ baseline characteristics*

	Usual care (n = 91)	Intervention group (n = 89)
Sample characteristics		
Age, mean ± SD years	69.87 ± 8.68	69.50 ± 9.17
Comorbidities, mean ± SD	1.65 ± 0.85	1.77 ± 0.97
IMD, mean ± SD	6.43 ± 2.80	6.68 ± 2.64
Female	49 (54)	50 (56)
White	88 (99)	84 (95)
Lives alone	23 (26)	28 (32)
Married	60 (67)	54 (61)
Retired	64 (72)	59 (67)
Education†	65 (71)	57 (64)

* Values are the number (%) unless indicated otherwise. IMD = Index of Multiple Deprivation.

† Education included those participants who left before or at school‐leaving age (trial education question did not specify age).

### Resource use, costs, and QALYs

In total, 98 physical therapy classes were carried out as part of the trial. The average length of time of each class was 100 minutes; no class was shorter than 90 minutes, and included physical therapists’ time to set up, clear up, and write patients’ notes. Sixty‐nine of the 89 patients (78%) who were randomized to receive the intervention attended 4 or more classes, and 42 patients (47%) attended all 6 classes. All 89 patients who were randomized to receive the intervention attended at least 1 class. No patients assigned to the control arm received any of the intervention classes. On average, 5.46 patients attended each class. The intervention cost, on average, was £179 (95% CI £108, £250) per patient offered the physical therapy intervention.

Table 2 shows adjusted cost components and outcomes by trial arm, including imputed data for the 12 months after primary TKR surgery. Code for the imputation model can be found in Supplementary Table 1, available on the *Arthritis Care & Research* website at http://onlinelibrary.wiley.com/doi/10.1002/acr.24903. The cost drivers for this trial were the costs of additional physical therapy, which were larger for the intervention group. This reflects the fact that more patients in the intervention arm sought additional physical therapy, as reported in Table 2 of the clinical results of the study by Lenguerrand et al (10). The mean NHS + PSS costs for the year postsurgery per patient were £1,740 (95% CI –£742, £4,221) in the intervention group, compared with £1,394 (95% CI –£780, £3,568) in the usual care group, representing an additional £346 (95% CI £38, £653) per patient in the intervention arm.

**Table 2:** Costs and outcomes by trial arm and by perspective over 12 months*

	Usual care (n = 91)		Intervention group (n = 89)		
Costs	No. of patients using resource	Mean ± SD cost per patient	No. of patients using resource	Mean ± SD cost per patient	Difference, mean ± SD
NHS + PSS		£1,394 ± £1,109†		£1,740 ± £1,266†	£346 ± £157†
PT intervention classes	0	–	89	£179 ± £36	£179
Additional physical therapy	66	£139 ± £293	83	£277 ± £323	–£138 ± £30
Other therapies	37	£80 ± £199	35	£75 ± £156	£5 ± –£43
Hospital readmission	19	£192 ± £874	11	£125 ± £570	£66 ± –£305
Outpatient or AE visit	59	£127 ± £191	54	£129 ± £239	–£2 ± £48
Special orthopedic equipment	77	£62 ± £62	79	£109 ± £488	–£47 ± £426
Medication	80	£22 ± £33	66	£41 ± £143	–£19 ± £110
Other community health and social services use	76	£77 ± £117	76	£91 ± £152	–£14 ± £35
Societal		£3,418 ± £1,266†		£3,826 ± £1,445†	£407 ± £179†
Paid time off	27	£146 ± £492	23	£193 ± £532	–£47 ± £40
Unpaid time off	56	£38 ± £85	52	£35 ± £87	£3 ± £3
Informal care	57	£26 ± £44	52	£18 ± £29	£8 ± –£14
Outcomes‡					
QALYs	–	0.665 ± 0.219	–	0.716 ± 0.240	0.051 ± 0.021
Utility at baseline	–	0.466 ± 0.248	–	0.411 ± 0.269	–0.055 ± 0.021
Utility at 12 months	–	0.730 ± 0.232	–	0.749 ± 0.241	0.019 ± 0.009
LEFS score	–	48.22 ± 17.58	–	52.85 ± 20.03	4.64 ± 2.45
LEFS score at baseline	–	28.59 ± 14.74	–	25.39 ± 14.58	–3.20 ± –0.16
LEFS score at 12 months	–	53.29 ± 17.53	–	55.79 ± 18.48	2.50 ± 0.95

* National Health System (NHS) + Personal Social Services (PSS) and societal cost totals and outcomes were estimated adjusting for sex, age, hospital site, comorbidities, and baseline outcomes in seemingly unrelated regression baseline models complete data using 60 imputed data sets. AE = accident or emergency; LEFS = Lower Extremity Functional Scale. Total societal costs include all NHS + PSS costs plus the additional categories of “paid time off,” “unpaid time‐off,” and informal care. Individual cost categories were imputed for missing data using the same imputation model, but mean and SDs were not adjusted using regression analysis. PT = physical therapy; QALY = quality‐adjusted life year.

† Values are the sum of all subcategories as estimates with imputed data and regression analysis; therefore, they do not equate to the arithmetic sum of the subcategories.

‡ Outcomes values for the usual care and intervention groups are the mean ± SD benefit.

The QALY benefits were also 0.0506 higher (95% CI 0.009, 0.09) in the intervention group (mean QALY gain over 12 months 0.7156 [95% CI 0.244, 1.18]) compared with the usual care group (mean QALY gain 0.6650 [95% CI 0.235, 1.09]). This equates to ~18.5 additional days in full health for patients in the intervention arm.

### Cost–utility and cost‐effectiveness base case results

The mean INMB statistic was £665 (95% CI £139, £1,191), for a WTP threshold of £20,000 per QALY (Table 3). This means that the physical therapy group intervention costs, on average, an additional £6,842 per QALY gained compared with usual care, from an NHS + PSS perspective. From a societal perspective, the costs accruing from both intervention and usual care arms were higher, and the mean INMB statistic was £407 (95% CI £56, £758). The ICER was £8,003 per QALY gained, which, although higher than in our primary analysis, still sits comfortably under the £20,000 threshold used by NICE.

**Table 3:** Base case results: cost utility analysis (CUA) and cost‐effectiveness analysis (CEA)*

	NHS + PSS perspective	Societal perspective
CUA		
ICER (£/QALY)	£6,842	£8,003
Prob. cost‐effectiveness (%)	91.74	89.41
INMB (95% CI)†	£665 (£139, £1,191)	£407 (£56, £758)
CEA		
ICER (£/LEFS score)	£74.66	£87.10

* Base case models adjusted for age, sex, comorbidities, and hospital site. 95% CI = 95% confidence interval; ICER = incremental cost‐effectiveness ratio; INMB = incremental net monetary benefit; prob. = probability. See Table 2 for other definitions.

‡ Measured at a willingness‐to‐pay threshold of £20,000 for a QALY.

The mean difference in LEFS score between arms was below the minimum clinically important difference. The intervention costs an additional £75 per unit LEFS score gained from an NHS + PSS perspective, and £87 per unit LEFS score gained from a societal perspective.

The correlation coefficients in the SUR models were negative for both the relationship between costs and QALYs (–0.0123 for the NHS + PSS perspective; –0.109 for the societal perspective) and costs and LEFs scores (−0.328 and –0.336, respectively). This means that patients with high health care or societal costs were those with worse health outcomes.

**Figure 1:**
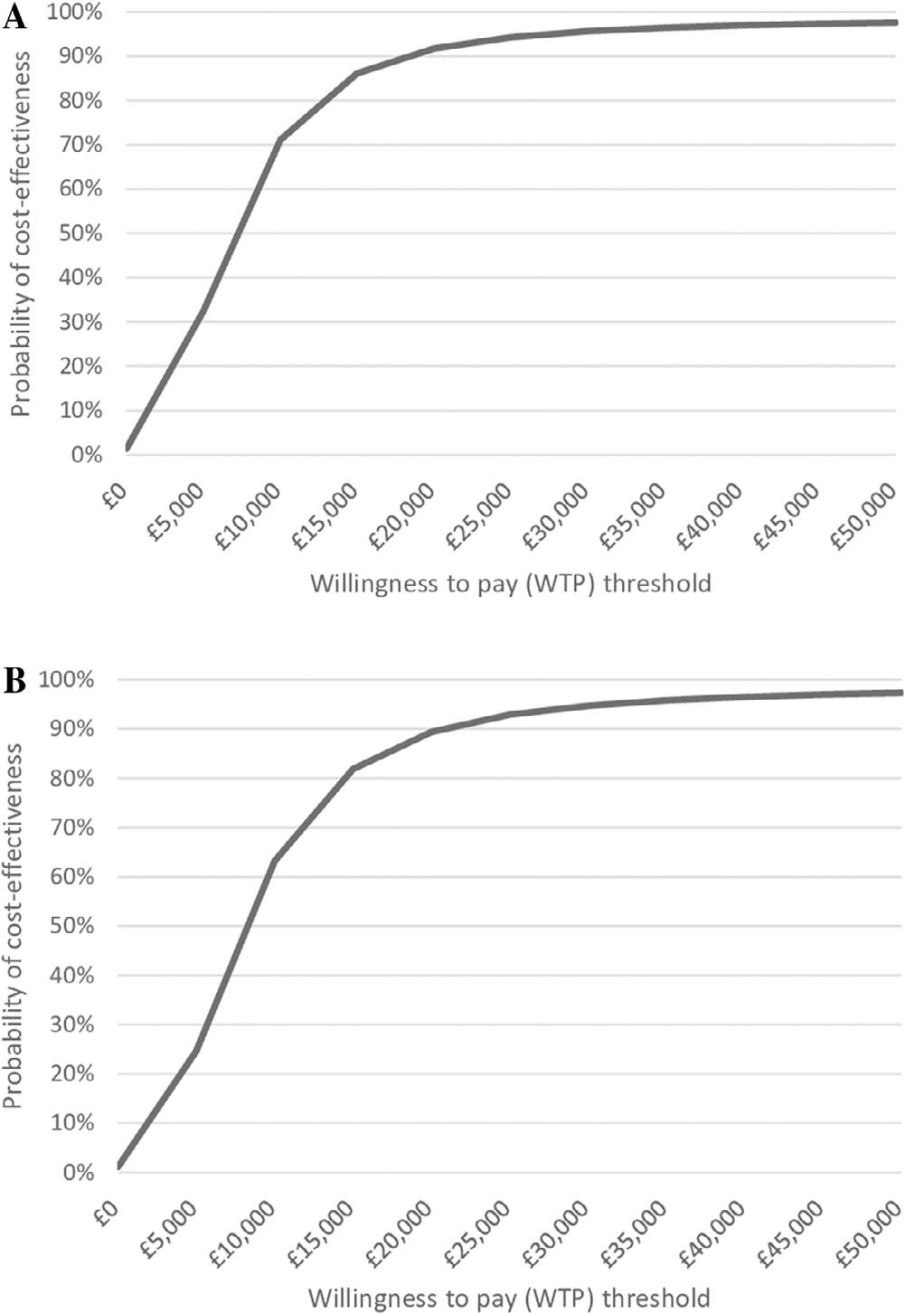
Cost‐effectiveness acceptability curve showing the probability that the intervention is cost‐ effective versus usual care at different values of the societal willingness‐to‐pay threshold for a quality‐adjusted life year, from a National Health System plus Personal Social Services perspective (**A**) and a societal perspective (**B**).

Figure 1 demonstrates the probability of the intervention being cost‐effective for WTP thresholds varying from £0 to £50,000 per QALY gained in cost‐effectiveness acceptability curves. At £20,000 per QALY gained, the intervention is 91.74% likely to be cost‐effective from an NHS + PSS perspective and 89.41% likely to be cost effective from the societal perspective.

### Sensitivity analysis results

The sensitivity analysis to our statistical models yielded slightly higher costs and QALYs. From an NHS + PSS perspective, the costs associated with the intervention and usual care arms were £2,182 and £1,866 on average, respectively, and from a societal perspective, these were £4,014 and £3,671 on average, respectively. However, the mean difference in outcomes (QALYs and LEFS) between arms was also greater, resulting in higher mean INMBs (£769 from an NHS + PSS perspective; £745 from a societal perspective) and smaller ICERs (Table 4).

**Table 4:** Sensitivity analyses: using models type 2 specification, optimistic and pessimistic scenarios, complete case analyses*

	NHS + PSS perspective	Societal perspective
Models type 2		
ICER (£/QALY)	£5,819	£6,299
Prob. cost‐effectiveness, %	95.22	94.14
INMB (95% CI)	£769 (£291, £1,247)	£745 (£308, £1,183)
Optimistic scenario		
ICER (£/QALY)	£4,914	£6,086
Prob. cost‐effectiveness, %	94.42	92.62
INMB (95% CI)	£763 (£237, £1,289)	£708 (£225, £1,191)
Pessimistic scenario		
ICER (£/QALY)	£12,960	£14,084
Prob. cost‐effectiveness, %	77.11	73.10
INMB (95% CI)	£365 (–£169, £882)	£301 (–£181, £783)
Complete case		
ICER (£/QALY)	£9,282	£9,410
Prob. cost‐effectiveness, %	76.01	75.95
INMB (95% CI)	£419 (–£195, £1,034)	£428 (–£149, £1,007)

* Incremental net monetary benefit statistics (INMB) (95% confidence intervals [95% CIs]) were measured at a willingness‐to‐pay threshold of £20,000 per quality‐adjusted life year (QALY). ICER = incremental cost‐effectiveness ratio; NHS + PSS = National Health System + Personal Social Services; prob. = probability.

The differences in costs from the NHS + PSS and societal perspectives are, on average, smaller (£248 and £309) when classes run at capacity, with 12 participants per session in the optimistic scenario, compared with the base case. The differences are greater (£655 and £716) with only 2 participants per class in the pessimistic scenario, for both perspectives on costs. Even in the pessimistic scenario, the probability that the intervention is cost effective is above 70% when the society WTP threshold £20,000 per QALY.

Our complete case analysis included 108 patients with complete information prior to imputation. Our findings are more conservative, but the probability that the intervention is cost‐effective at the £20,000 WTP threshold is still higher than 75%, for both perspectives.

## DISCUSSION

The novel group‐based outpatient physical therapy 6 weeks after TKR costs an incremental £6,842 per QALY gained from an NHS + PSS perspective and an incremental £8,003 per QALY gained from a societal perspective. The additional physical therapy delivered in the ARENA intervention costs, on average, £179 per patient. In the year following surgery, care in the intervention group cost (on average) £346 more per patient than usual care, as patients in the intervention group sought additional physical therapy to the intervention, which is already more than usually offered in standard care. The QALY gains associated with the intervention are on average 0.0506 (95% CI 0.009, 0.09) higher, corresponding to 18.5 additional days of full health over the year for patients in the intervention group. This group‐based intervention is likely to be a cost‐effective treatment from both an NHS + PSS perspective and a societal perspective when compared with usual care alone for a society WTP threshold of £20,000 per QALY gained, and our findings were robust to model specification and different scenarios of patient uptake across the wider NHS.

The ARENA study was a large randomized controlled trial in the clinical rehabilitation literature, and included an economic evaluation, which allowed us to estimate whether spending additional resources to provide this intervention would be an efficient use of resources. By including a societal perspective on costs, we are also considering the burden of the intervention on patients, their careers, and society.

The ARENA intervention is a novel intervention developed by the study's physical therapy team. It is a short intervention, delivered to all patients “early” at 6 weeks postsurgery, which is an improvement on the current practice of delivering therapy on an as‐needed basis. It is delivered in a group setting, where physical therapists can run a weekly group class and patients can enter and leave the group at will. This setting makes the logistics of delivery easy to organize and cost‐effective for hospitals to provide. The exercises in the class are tailored to each patient, and thus patients can undertake a tailored intervention within a group setting. We found that even when the class size is only 2 patients per class, the intervention's incremental cost‐effectiveness ratio is still less than £20,000 per QALY gained and is more than 70% likely to be cost‐effective. We observed that patients in the intervention arm received more additional physical therapy. We expected that offering physical therapy to all patients in the ARENA intervention would substitute prescribed additional therapy on a “needed” basis; instead, we found it induced demand for additional prescribed therapy in the intervention group. We expected patients in the usual care group to seek further treatment to supplement their usual care, due to participating in trial. Although this may have happened, it was surpassed by the additional therapy sought by patients in the intervention group. The improvement of mean ± SD 0.051 ± 0.21 QALY gains observed at 1 year is higher than QALY gains observed in some other trials in TKR (30, 31, 32), but evidence is lacking on what constitutes a meaningful difference for QALY gains measured by the EQ‐5D‐5L instrument and valued using a crosswalk from the 3‐level values. The ARENA intervention is therefore cost‐effective in relation to this observed QALY gain and for a society WTP threshold of £20,000 per QALY gained. The improvement in LEFS score was too small to determine the cost‐effectiveness in relation to the primary clinical outcome.

All outcome and resource‐use data were collected from patient‐completed questionnaires at follow‐up periods, which allow for a wider perspective on costs to be taken but are prone to recall bias and missing data. However, our complete case analysis results were consistent with the imputed data findings. Our findings may not be generalizable in settings where there is no availability of gym space and/or staff time to deliver the intervention. Despite the relatively large sample size of this trial, we did not compute a sample size for the economic results, and they may be underpowered.

In October 2019, NICE started a consultation for further evidence on postoperative rehabilitation of joint replacement, including of the knee (33). Our results suggest that group‐based physical therapy classes are cost‐effective for a society WTP threshold of £20,000 per QALY gained and increase health‐related QoL by ~18.5 additional days in full health and therefore have the potential to contribute to future clinical guidelines. Other studies have already demonstrated the cost‐effectiveness of group‐based physical therapy for treatment of knee arthritis (34, 35, 36), but these studies were for interventions prior to knee replacement surgery, and the findings were based mostly on lower costs of the intervention, with very small increments in QoL. More recently, a study compared a home‐based rehabilitation program with traditional one‐to‐one physical therapy after partial knee replacement and TKR and found no evidence that the home‐based program led to great improvements in function or QoL (30). Our study provides new evidence that short‐term, group‐based physical therapy classes may be cost‐effective alternatives to rehabilitation following TKR, largely due to improvements in QoL. We present evidence on potentially efficient intervention available to all patients after TKR, which, if implemented, may reduce inequalities in access to care for underserved populations in TKR.

In conclusion, we found that group‐based outpatient physical therapy classes delivered 6 weeks after surgery in addition to usual NHS care is a cost‐effective clinical rehabilitation option for patients following primary TKR for a society WTP threshold of £20,000 per QALY gained. It costs an additional £346 per patient to the health care provider in the year following surgery and leads to increases in QALYs and small, nonmeaningful, improvements in function. Our findings were robust in a range of sensitivity analyses and when taking a societal perspective on costs.

## AUTHOR CONTRIBUTIONS

All authors were involved in drafting the article or revising it critically for important intellectual content, and all authors approved the final version to be submitted for publication. Dr. Marques had full access to all of the data in the study and takes responsibility for the integrity of the data and the accuracy of the data analysis.

### Study conception and design

Wylde, Artz, Blom, Marques.

### Acquisition of data

Wylde, Sanderson, Lenguerrand, Marques.

### Analysis and interpretation of data

Barbosa, Thorn, Marques.

## Supporting information


**Disclosure Form**: Click here for additional data file.

**Table S1** 
