# Supplementary material for: Cost‐Effectiveness of Group‐Based Outpatient Physical Therapy After Total Knee Replacement: Results From the Economic Evaluation Alongside the ARENA Multicenter Randomized Controlled Trial
Source: Arthritis Care Res (Hoboken). 2022 Sep 12;74(12):1970–7. doi: 10.1002/acr.24903 (PMC10087974; doi:10.1002/acr.24903)
Supplement: Supplementary file 2 — Table S1 [file ACR-74-1970-s001.docx]

| **Cost-effectiveness of group-based outpatient physical therapy after total knee replacement: results from the economic evaluation alongside the ARENA multicentre randomised controlled trial**  Estela C. Barbosa^1^ PhD, Vikki Wylde^1,2^ PhD, Joanna Thorn^3^ PhD, Emily Sanderson^3^ MSc, Erik Lenguerrand^1^ PhD, Neil Artz^4^ PhD, Ashley W. Blom^1,2^ PhD, Elsa M. R. Marques*^1,2^  Supplementary materials: STATA Coding  **Coding for Seemingly Unrelated Regression**  //Regress using seemingly unrelated regression (SUR) //  // Primary analysis - NHS+PSS //  xi: mi estimate, cmdok: sureg ( totalNHSPSScost treatmentgroup site age sex comorbidity imd married alone white work education) /*  */(totalQALY treatmentgroup utility_baseline site age sex comorbidity pre_op_LEFS_0 imd married alone white work education), corr  //Probability of cost-effectiveness using coefficients from SUR  matrix beta = e(b_mi) // extract coefficients  matrix vari = e(V_mi) // extract variance-covariance matrix  scalar QD = beta[1,13] // difference in QALYs  scalar CD = beta[1,1] // difference in costs  scalar varQD= vari[13,13] // variance for QALYs  scalar varCD = vari[1,1] // variance for costs  scalar cov = vari[13,1] // covariance  di CD/QD // ICER  di normal((20000*QD-CD)/sqrt((20000)^2 * varQD + varCD - 2*20000*cov)) // Probability of cost-effective  di ((QD*20000)-CD) // NMB  // Secondary Analysis - Societal Costs //  xi: mi estimate, cmdok: sureg ( totalcost treatmentgroup site age sex comorbidity imd married alone white work education) /*  */(totalQALY treatmentgroup utility_baseline site age sex comorbidity pre_op_LEFS_0 imd married alone white work education), corr  //Probability of cost-effectiveness using coefficients from SUR  matrix beta = e(b_mi) // extract coefficients  matrix vari = e(V_mi) // extract variance-covariance matrix  scalar QD = beta[1,12] // difference in QALYs  scalar CD = beta[1,1] // difference in costs  scalar varQD= vari[12,12] // variance for QALYs  scalar varCD = vari[1,1] // variance for costs  scalar cov = vari[12,1] // covariance  di CD/QD // ICER  di normal((20000*QD-CD)/sqrt((20000)^2 * varQD + varCD - 2*20000*cov)) // Probability of cost-effectiveness  di ((QD*20000)-CD) // NMB  /// LEFS analysis - NHS+PPS costs ///  xi: mi estimate, cmdok: sureg ( totalNHSPSScost treatmentgroup site age sex comorbidity imd married alone white work education) /*  */(post_op_LEFS_12 treatmentgroup pre_op_LEFS_0 site age sex comorbidity imd married alone white work education), corr  //ICER using coefficients from SUR //  matrix beta = e(b_mi) // extract coefficients  matrix vari = e(V_mi) // extract variance-covariance matrix  scalar QD = beta[1,12] // difference in QALYs  scalar CD = beta[1,1] // difference in costs  scalar varQD= vari[12,12] // variance for QALYs  scalar varCD = vari[1,1] // variance for costs  scalar cov = vari[12,1] // covariance  di CD/QD // ICER  /// LEFS analysis - societal costs ///  xi: mi estimate, cmdok: sureg ( totalcost treatmentgroup site age sex comorbidity imd married alone white work education) /*  */ (post_op_LEFS_12 treatmentgroup pre_op_LEFS_0 site age sex comorbidity imd married alone white work education), corr  //ICER using coefficients from SUR //  matrix beta = e(b_mi) // extract coefficients  matrix vari = e(V_mi) // extract variance-covariance matrix  scalar QD = beta[1,12] // difference in QALYs  scalar CD = beta[1,1] // difference in costs  scalar varQD= vari[12,12] // variance for QALYs  scalar varCD = vari[1,1] // variance for costs  scalar cov = vari[12,1] // covariance  di CD/QD // ICER  **Coding for Sensitivity Analysis**  // Primary analysis - NHS+PSS //  xi: mi estimate, cmdok: sureg ( totalNHSPSScost treatmentgroup site age sex comorbidity) (totalQALY treatmentgroup utility_baseline site age sex comorbidity pre_op_LEFS_0), corr  //Probability of cost-effectiveness using coefficients from SUR  matrix beta = e(b_mi) // extract coefficients  matrix vari = e(V_mi) // extract variance-covariance matrix  scalar QD = beta[1,7] // difference in QALYs  scalar CD = beta[1,1] // difference in costs  scalar varQD= vari[7,7] // variance for QALYs  scalar varCD = vari[1,1] // variance for costs  scalar cov = vari[7,1] // covariance  di CD/QD // ICER  // Cost-effectiveness acceptability curve //  di normal((0.0000001*QD-CD)/sqrt((0.00001)^2 * varQD + varCD - 2*0.000001*cov))  di normal((5000*QD-CD)/sqrt((5000)^2 * varQD + varCD - 2*5000*cov))  di normal((10000*QD-CD)/sqrt((10000)^2 * varQD + varCD - 2*10000*cov))  di normal((15000*QD-CD)/sqrt((15000)^2 * varQD + varCD - 2*15000*cov))  di normal((20000*QD-CD)/sqrt((20000)^2 * varQD + varCD - 2*20000*cov))  di normal((25000*QD-CD)/sqrt((25000)^2 * varQD + varCD - 2*25000*cov))  di normal((30000*QD-CD)/sqrt((30000)^2 * varQD + varCD - 2*30000*cov))  di normal((35000*QD-CD)/sqrt((35000)^2 * varQD + varCD - 2*35000*cov))  di normal((40000*QD-CD)/sqrt((40000)^2 * varQD + varCD - 2*40000*cov))  di normal((45000*QD-CD)/sqrt((45000)^2 * varQD + varCD - 2*45000*cov))  di normal((50000*QD-CD)/sqrt((50000)^2 * varQD + varCD - 2*50000*cov))  // Secondary Analysis - Societal Costs //  xi: mi estimate, cmdok: sureg ( totalcost treatmentgroup site age sex comorbidity) (totalQALY treatmentgroup utility_baseline site age sex comorbidity pre_op_LEFS_0), corr  //Probability of cost-effectiveness using coefficients from SUR  matrix beta = e(b_mi) // extract coefficients  matrix vari = e(V_mi) // extract variance-covariance matrix  scalar QD = beta[1,7] // difference in QALYs  scalar CD = beta[1,1] // difference in costs  scalar varQD= vari[7,7] // variance for QALYs  scalar varCD = vari[1,1] // variance for costs  scalar cov = vari[7,1] // covariance  di CD/QD // ICER  // Cost-effectiveness acceptability curve //  di normal((0.0000001*QD-CD)/sqrt((0.00001)^2 * varQD + varCD - 2*0.000001*cov))  di normal((5000*QD-CD)/sqrt((5000)^2 * varQD + varCD - 2*5000*cov))  di normal((10000*QD-CD)/sqrt((10000)^2 * varQD + varCD - 2*10000*cov))  di normal((15000*QD-CD)/sqrt((15000)^2 * varQD + varCD - 2*15000*cov))  di normal((20000*QD-CD)/sqrt((20000)^2 * varQD + varCD - 2*20000*cov))  di normal((25000*QD-CD)/sqrt((25000)^2 * varQD + varCD - 2*25000*cov))  di normal((30000*QD-CD)/sqrt((30000)^2 * varQD + varCD - 2*30000*cov))  di normal((35000*QD-CD)/sqrt((35000)^2 * varQD + varCD - 2*35000*cov))  di normal((40000*QD-CD)/sqrt((40000)^2 * varQD + varCD - 2*40000*cov))  di normal((45000*QD-CD)/sqrt((45000)^2 * varQD + varCD - 2*45000*cov))  di normal((50000*QD-CD)/sqrt((50000)^2 * varQD + varCD - 2*50000*cov)) |
| --- |
